# Coexist or eliminate? Antibody-based functional silencing versus eradication in gut microbiome- targeted therapy

**DOI:** 10.3389/fimmu.2026.1903103

**Published:** 2026-07-15

**Authors:** Sherif A. El-Kafrawy, Ayman T. Abbas, Abdelrahman S. El-Kafrawy, Mai M. El-Daly, Esam I. Azhar

**Affiliations:** 1 Special Infectious Agents Unit-Biosafety Level 3 (BSL3), King Fahd Medical Research Center, King Abdulaziz University, Jeddah, Saudi Arabia; 2 Department of Medical Laboratory Sciences, Faculty of Applied Medical Sciences, King Abdulaziz University, Jeddah, Saudi Arabia; 3 Faculty of Medicine, Menoufia University, Shebin El-Koam, Egypt

**Keywords:** antibodies, functional silencing, IgY, MAbs, microbiome, precision medicine

## Abstract

**Background:**

Dominant anti-infective strategies equate therapeutic success with pathogen eradication, yet in the gut most clinically relevant “pathogens” are pathobionts that cause disease only under specific ecological conditions. Antibiotics resolve infection but decimate commensal communities and select for resistance.

**Scope and approach:**

We review antibody-based interventions, native mucosal antibodies (IgA, IgM), monoclonal antibodies, and immunoglobulin Y (IgY), as mechanistic test cases for a functional silencing paradigm, in which pathobiont virulence is attenuated through non-bactericidal mechanisms while preserving community architecture.

**Key findings:**

Drawing on randomized trial data (bezlotoxumab in *Clostridioides difficile* recurrence prevention, MODIFY I/II, n = 2,655), preclinical and early clinical IgY studies in enteric infections, and long-term IgY deployment in aquaculture, we find that functional silencing delivers durable benefit when disease is driven by discrete virulence factors. However, evolutionary risks, including phase variation, conformational switching, and the theoretical framework of “imperfect immunity”, identify conditions under which retained pathobionts may re-emerge as threats.

**Conclusions:**

Eradication and functional silencing are best understood as complementary strategies whose optimal deployment depends on host immune status, barrier integrity, and the nature of pathobiont virulence. Defining the boundary between safe coexistence and evolutionary rebound constitutes the field’s central unresolved challenge.

## 1 Introduction

### 1.1 From eradication to ecosystem management

Modern anti-infective strategies rest on a simple premise: pathogens must be eliminated (1–3). In the gut, however, this eradication paradigm collides with the reality that most clinically relevant “pathogens” are long-term residents of a densely populated microbial ecosystem (4–6). Members of Enterobacteriaceae, *Enterococcus*, and other pathobionts persist in healthy hosts and cause disease only under specific ecological or immunological conditions (4–6). A central question follows: must we remove these organisms entirely, or can we restore conditions under which they remain quiescent co-residents?

The human gastrointestinal tract harbors approximately 10^13–^10^14^ microorganisms, collectively encoding 150-fold more genes than the host genome (7, 8). This community, dominated by the phyla Firmicutes (Bacillota) and Bacteroidetes (Bacteroidota) with substantial representation from Actinobacteria, Proteobacteria, and Verrucomicrobia, exists in dynamic equilibrium with the host (7, 9). These microbes function as a metabolically active organ, co-evolved with mammals to regulate processes spanning immunity and metabolism to gut-brain communication (8, 10).

### 1.2 Microbiome-mediated homeostasis and dysbiosis

The gut microbiome sustains host physiology through biochemical transformations inaccessible to human enzymatic repertoires (11). Anaerobic fermentation of dietary fibers generates short-chain fatty acids (SCFAs), with butyrate serving as both the primary colonocyte energy substrate and an epigenetic regulator of inflammatory gene expression through histone deacetylase inhibition and GPR43/GPR109A signaling, inducing T_reg_ differentiation, maintaining tight junction integrity, and suppressing NF-κB-mediated inflammation (11, 12). Additional metabolic circuits, bile acid biotransformation via FXR and TGR5 signaling (13, 14) and tryptophan-derived AhR ligands promoting IL-22 production and barrier fortification (15, 16), further calibrate host immunity and metabolism. These immunomodulatory functions emerge during developmental windows: commensal-driven Th17 and T_reg_ maturation is essential for mucosal immune homeostasis, as demonstrated by the impaired immune ontogeny of germ-free animals (17–19).

When this equilibrium is perturbed, dysbiosis reflects disruptions in the host-provided intestinal environment, not merely a shift in microbial composition (5, 20). During homeostasis, colonocyte-mediated oxygen consumption maintains luminal anaerobiosis, selectively favoring obligate anaerobes while restricting Proteobacteria (20, 21). Barrier deterioration, through genetic susceptibility, inflammatory insults, or antibiotic-induced community collapse, allows oxygen and alternative electron acceptors to reach the lumen, conferring growth advantages to pathobionts whose expansion amplifies inflammation through LPS translocation and TLR4 signaling (20–23). The resulting pattern, reduced alpha-diversity, depletion of SCFA producers, and enrichment of Enterobacteriaceae, is consistently observed across inflammatory bowel disease, metabolic syndrome, and systemic autoimmunity (24, 25).

### 1.3 Limitations of current approaches and the case for antibody-based strategies

Broad-spectrum antibiotic administration reduces microbial diversity by 25–50%, with reconstitution requiring months to years and potentially permanent loss of keystone taxa (26). Antibiotics damage beneficial commensals and compromise colonization resistance. They also thin the mucus layer, reduce antimicrobial peptide secretion, and promote the selection of resistant organisms, ecological disruptions that can persist for months to years (1–3, 26). Alternative strategies face their own limitations. Probiotics and fecal microbiota transplantation (FMT), while promising for specific indications such as recurrent *C. difficile* infection, exhibit variable engraftment, lack antigenic precision, and carry safety concerns (3, 26–28). Dietary approaches operate through broad community-level perturbations with responses varying by baseline enterotype (29–32). Two limitations recur across all modalities: lack of taxon- and function-level specificity, and poor control over ecological side-effects.

The field has not yet fully exploited the host’s own evolved toolkit for precise, non-sterilizing control of microbiota. In the healthy gut, immense quantities of secretory IgA and IgM contain and constrain microbial populations without sterilizing the lumen, preferentially targeting Proteobacteria while largely sparing dominant Firmicutes and Bacteroidetes (4, 6, 33–38). Pathobionts are not routinely eradicated; they are contained and behaviorally constrained. This observation motivates a shift from “How can we better eliminate harmful microbes?” to “How can we therapeutically reinforce the host’s existing containment systems to keep pathobionts in a disarmed, commensal-like state?” Antibody-based approaches are uniquely positioned to test this functional silencing paradigm.

### 1.4 The antibody-microbiome interface: an evolutionary precedent

Nature provides a compelling model for precision microbiome modulation through antibody-mediated regulation (27, 35). The intestinal mucosa constitutively secretes large quantities of SIgA and SIgM, which together coat a substantial fraction of the gut microbiota without sterilizing the lumen (4, 36, 37). These mucosal antibodies shape community composition through non-inflammatory mechanisms: limiting bacterial motility and epithelial penetration while spatially segregating microbes from the epithelium. SIgA also promotes retention of beneficial taxa through “immune inclusion” and strengthens colonization resistance against invading pathogens (4, 35, 37–39).

Much of this surveillance is pre-emptive. A substantial fraction of microbiota-reactive IgA comprises natural polyreactive antibodies with innate-like specificity, arising independently of exogenous antigenic stimulation, and similar IgA specificities are detectable even in germ-free mice (33, 34). The adaptive immune system has co-opted innate-like recognition modules to constrain microbiota members with higher pathogenic potential before disease develops. The molecular mechanisms of SIgA and SIgM that underpin these functions are detailed in Section 2.1.

Mucosal antibodies are ecological sculptors that modulate microbial behavior, spatial organization, and immune visibility without indiscriminate killing (4, 34, 35, 37). This evolutionary precedent supports the notion that enduring host-microbiome mutualism is achieved through co-existence with control, not through repeated eradication.

### 1.5 Therapeutic antibody platforms

Three major antibody platforms extend this evolutionary logic to therapeutic contexts (40–42):

Monoclonal antibodies (mAbs) target surface antigens, adhesins, toxins, or metabolic enzymes, enabling both direct pathogen neutralization and functional reprogramming (40, 43–45).

Immunoglobulin Y (IgY), produced in avian species and harvested non-invasively from egg yolk, offers phylogenetically distant, non-inflammatory antibodies that do not fix mammalian complement or engage Fc receptors, making them well-suited for chronic enteral administration (40, 41, 44–48).

Engineered IgA harnesses the evolved properties of mucosal IgA, polymeric structure, secretory component association, and mucus anchoring, while incorporating defined therapeutic specificities (4, 35, 49).

Antibody binding to adhesins, flagella, toxins, quorum-sensing molecules, or key enzymes attenuates virulence, limits mucosal access, and reshapes host-microbe signaling without eradicating the organism from its niche (3, 37, 40). Such interventions could resolve disease-driving interactions while preserving colonization resistance, SCFA production, and community architecture, addressing the ecological vulnerabilities created by antibiotics. The following sections dissect how different antibody classes operate at the host-microbiome interface and evaluate their efficacy and limitations as instruments of functional silencing versus eradication.

## 2 Antibody platforms and target selection

### 2.1 Native mucosal antibodies: IgA and IgM

Secretory IgA constitutes the dominant immunoglobulin in the intestinal lumen, with humans secreting gram quantities annually, representing more than 80% of total antibody-producing plasma cells in the lamina propria (4, 37). Intestinal IgA exists primarily as dimers joined by the J-chain, which stabilizes dimeric structure and provides a ligand for the polymeric immunoglobulin receptor (pIgR) on epithelial cells (4, 50). Following transcytosis, pIgR undergoes proteolytic cleavage to release secretory IgA complexed with the secretory component, enhancing proteolytic stability and providing mucus-anchoring sites through glycan interactions (50, 51).

Single-cell repertoire analysis demonstrates selective enrichment for polyreactive IgA targeting bacterial glycans, particularly immunodominant epitopes on Proteobacteria, while largely sparing Firmicutes and Bacteroidetes (34). Such differential coating shapes microbiome composition by limiting bacterial motility via agglutination and “enchainment” of dividing bacteria, modulating bacterial gene expression, including virulence factors (52, 53), and facilitating niche colonization by beneficial commensals through secretory component-mediated mucus anchoring (54, 55).

Humans uniquely express two IgA subclasses (4). IgA2, enriched in the distal intestine, possesses a shorter hinge region conferring superior resistance to bacterial proteases, whereas IgA1 exhibits enhanced binding flexibility. IgA1 binds the high-affinity Fcα receptor I (FcαRI; CD89) on phagocytes, enabling pro-inflammatory responses upon penetration of IgA-coated bacteria into the mucosa (4, 56).

Secretory IgM provides a complementary layer of microbiome regulation, particularly in humans, where gut IgM-secreting plasma cells comprise 10–20% of total plasma cells (4, 36). Pentameric IgM translocates across epithelia via pIgR through J-chain-mediated interactions analogous to IgA. SIgM targets bacterial consortia dually coated by SIgA, with doubly-coated bacteria enriched in beneficial Firmicutes and exhibiting greater diversity than IgA-only coated populations (36). In IgA-deficient patients, SIgM partially compensates, though with limited binding to Enterobacteriaceae and incomplete restoration of microbiota diversity, underscoring non-redundant functions (36, 57).

### 2.2 Monoclonal antibodies

Monoclonal antibodies offer single-epitope specificity achievable through hybridoma technology or recombinant expression platforms (40, 43). Their therapeutic rationale in microbiome modulation centers on selective pathobiont targeting without collateral damage to commensals (58, 59). Landmark studies demonstrate that antibacterial mAbs directed against *Staphylococcus aureus* surface antigens, *Pseudomonas aeruginosa* lipopolysaccharide, and *C. difficile* toxins A/B achieve pathogen clearance while preserving microbiome diversity, in stark contrast to antibiotic-induced dysbiosis (58, 60).

Recent advances expand mAb targeting beyond structural antigens to include virulence factors and metabolic enzymes (40, 45). mAbs neutralizing adhesins (*FimH* from uropathogenic *E. coli*), capsular polysaccharides, and toxins implement functional silencing, preserving niche occupancy while attenuating pathogenic behaviors (44, 45). Additionally, mAbs targeting bacterial β-lactamases restore antibiotic susceptibility in resistant strains, enabling synergistic combination therapies (61).

### 2.3 Immunoglobulin Y

IgY, the functional equivalent of mammalian IgG in avian species, offers distinctive advantages rooted in phylogenetic divergence (41, 47). The approximately 300-million-year evolutionary separation between birds and mammals enables immunization of chickens against conserved bacterial epitopes that elicit weak responses in mammals due to immune tolerance (41, 62). Structurally, IgY (~180 kDa) differs from IgG (~150 kDa) through an additional constant domain (Cυ2) and absence of a hinge region, resulting in a rigid architecture that paradoxically enhances stability (46, 47).

The critical functional distinction lies in IgY’s inability to activate mammalian complement or bind mammalian Fc receptors, eliminating pro-inflammatory complications and enabling chronic oral delivery without inducing antibody-dependent cellular cytotoxicity (41, 48). IgY also exhibits favorable acid stability and protease resistance, retaining approximately 40% activity after eight hours of trypsin or chymotrypsin exposure (46, 62).

IgY production offers remarkable scalability: a single hen produces approximately 22–25 grams of total IgY annually, with 2–10% comprising antigen-specific antibodies, achievable through simple egg collection without terminal blood harvesting (62). Mechanistically, IgY modulates the microbiome through non-bactericidal pathways: inhibiting adhesion, agglutinating target bacteria, suppressing motility by binding flagella, inhibiting key enzymes (urease, β-lactamases), and neutralizing toxins (41, 62–64).

### 2.4 Target antigens

Rational antigen selection determines whether antibody-based interventions behave as eradication tools or instruments of functional silencing. The most promising targets for microbiome-targeted therapy control virulence, barrier disruption, or ecological behavior, rather than housekeeping functions required for survival.

Surface structural components: Outer membrane proteins critical for adherence and immune evasion in Gram-negative bacteria are attractive targets; mAbs and IgY against these antigens impair colonization by *E. coli* and *Salmonella* spp. without necessarily sterilizing the lumen (40, 41). Antibodies directed at LPS O-antigens neutralize endotoxin activity, reducing inflammatory signaling and systemic toxicity (64, 65).
Virulence factors: Adhesins including *FimH* and CFA/I are essential for epithelial attachment; antibodies against these structures block colonization and attenuate disease in preclinical and early clinical studies (66, 67). Anti-flagellin IgY reduces migration and invasion by *H. pylori* and *Salmonella*, limiting tissue access without eradicating luminal populations (41, 63). These interventions implement functional silencing, preventing close host contact while preserving niche occupancy and colonization resistance.
Secreted toxins and enzymes: Antibodies against cholera toxin, Shiga toxin, heat-labile and heat-stable enterotoxins, and *C. difficile* toxins A/B prevent pathogenesis without killing the producing bacteria, reducing diarrhea, epithelial damage, and recurrence risk (68–71). Urease blockade reduces *H. pylori* gastric colonization and pathology, while β-lactamase inhibition restores antibiotic susceptibility (61, 68, 69). Toxin- and enzyme-directed antibodies exemplify pure functional silencing, separating disease-causing activities from organism survival.

Multi-epitope targeting, combining antibodies against several antigens simultaneously, minimizes the risk of bacterial escape mutations and broadens coverage. Synergistic cocktails of mAbs and IgY are increasingly explored for robust efficacy and resistance prevention (72, 73).

## 3 Mechanisms of antibody-mediated microbiome modulation

**Figure 1:**
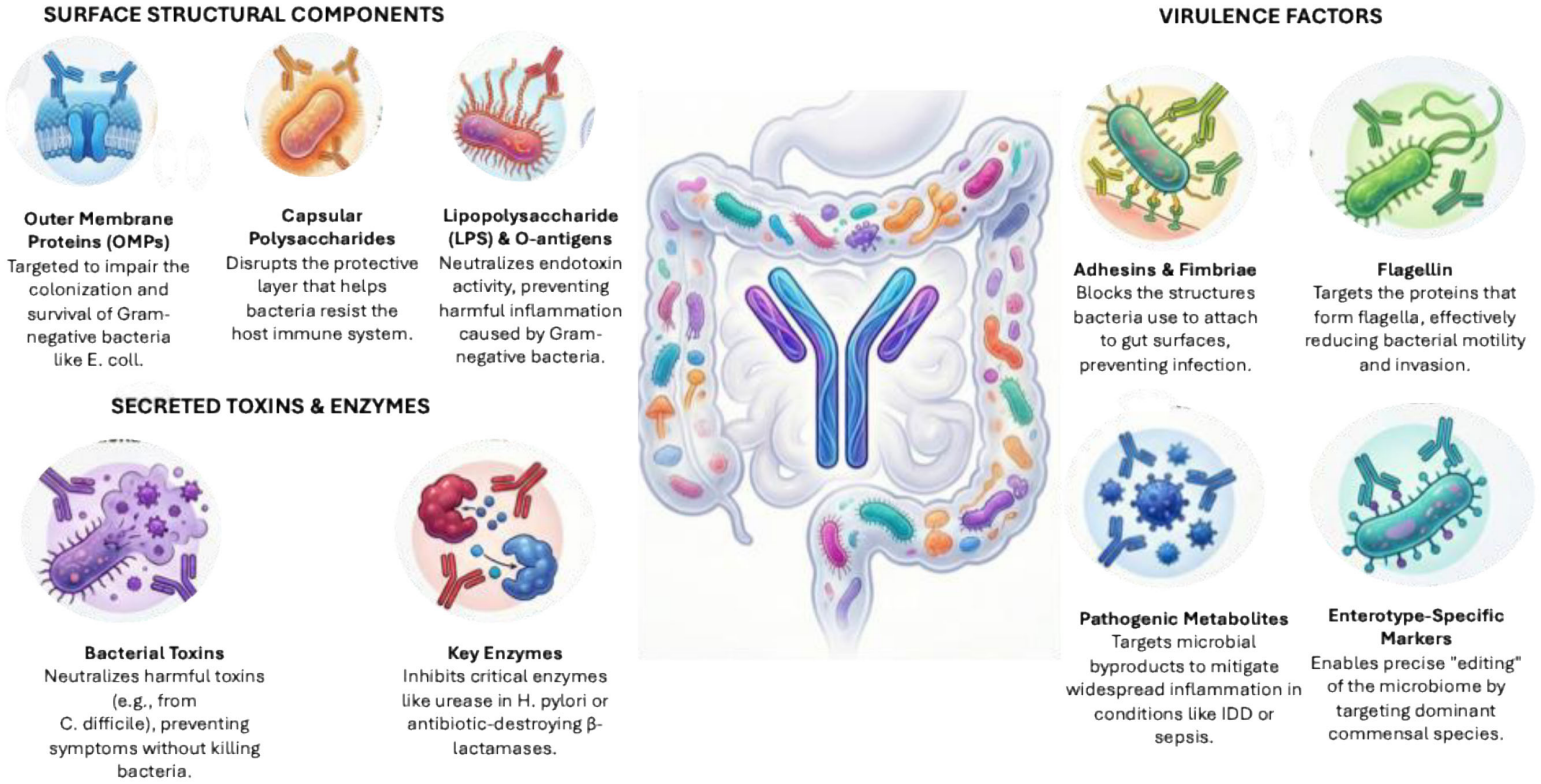
Targeting the microbiome: key antigens for antibody therapies. The success of antibody-based therapies like mAbs and IgY for microbiome engineering depends on selecting the right microbial target. These antigens fall into several functional categories, allowing for highly specific interventions that can neutralize pathogens or rebalance the gut ecosystem without the collateral damage of antibiotics.

Antibody-based interventions modulate the gut microbiome through specific, multi-level mechanisms distinct from the broad effects of conventional antimicrobials. At least five principal mechanisms underpin their efficacy (**Figure 1**).

### 3.1 Inhibition of pathogen adhesion and colonization

Both mAbs and IgY bind bacterial adhesins, fimbriae, and surface proteins, preventing initial epithelial attachment and limiting colonization by enteropathogens such as ETEC, *Salmonella*, and *H. pylori* (54, 67).

### 3.2 Pathogen agglutination and enhanced clearance

Polyvalent antibodies agglutinate target bacteria into large clusters efficiently removed by peristalsis, physically restricting microbial motility and reducing mucosal invasion without cytotoxicity or collateral damage to commensals (45, 63, 72).

### 3.3 Neutralization of toxins and metabolic enzymes

High-affinity neutralization of microbial toxins (*C. difficile* toxin, Shiga toxin, cholera toxin) and key enzymes (urease, β-lactamase) mitigates mucosal inflammation, prevents barrier disruption, and can restore antibiotic susceptibility, with negligible ecological disturbance (61, 71, 72).

### 3.4 Suppression of motility and quorum sensing

Antibody engagement with bacterial flagella or signaling molecules reduces motility and biofilm formation, suppressing coordinated expression of virulence factors and impeding pathobionts from exploiting inflammatory microenvironments (52, 74).

### 3.5 Immune modulation via selective microbial coating

Selective antibody coating, especially by SIgA, shapes immune recognition of gut microbes, tolerizing commensal populations and functionally silencing pathobionts by excluding them from close epithelial contact (70, 75, 76).

Collectively, these mechanisms yield pathogen-specific control with preservation of beneficial microbiota, a sharp contrast to the community disruption and resistance generation typical of antibiotic interventions.

## 4 Evidence from enteric infections and aquaculture

Antibody-based modulation of the gut microbiome has been explored across multiple contexts, though the depth of evidence varies substantially. We organize the following discussion by evidence maturity: applications supported by randomized clinical trial data (bezlotoxumab in *C. difficile*), those with preclinical efficacy and early human data (IgY in enteric infections, *H. pylori*), and those with extensive deployment in non-human settings (aquaculture). **Table 1** provides representative clinical trials.

**Table 1:** Representative clinical trials and studies illustrating antibody-based and related microbiome interventions.

Trial ID (phase)	Status	Registration date	Intervention	Primary microbiome/antibody endpoint
Antibody-based interventions				
NCT06973889	Adults with *H. pylori* infection	January 14, 2025	Oral IgY-enriched egg yolk preparation vs control	Pathogen-specific IgY targeting *H. pylori*; effects on gastric and gut microbiota load (93)
FP7 IgY-Pseudomonas (EU)	Cystic fibrosis patients at risk of *P. aeruginosa* colonization	NA*	Oral/topical anti-Pseudomonas IgY prophylaxis	IgY to prevent airway and gut colonization; microbiome and resistance endpoints (94)
NCT01241552/NCT01513239 (Phase III)	Adults with *C. difficile* infection on standard antibiotics	November 2010/June 2012	Bezlotoxumab (anti-toxin B mAb) ± actoxumab (anti-toxin A mAb)	CDI recurrence prevention; *post hoc* microbiome analyses (71)
Related microbiome modulation strategies				
NCT03353402	Anti-PD-1-refractory metastatic melanoma	November 2017	FMT from ICI responders + anti-PD-1 therapy	FMT-induced microbiome shift to restore response to checkpoint antibody (95)
NCT03341143	Metastatic melanoma, primary PD-1 resistance	November 2017	FMT + pembrolizumab	Donor-type microbiota engraftment; immune and metabolic reprogramming of tumor microenvironment (96)
NCT04758507	Renal cell carcinoma on ICIs	February 2021	FMT from ICI responders	Change in α/*β*-diversity and correlation with ICI outcomes (97)
NCT04130763	GI cancers failing anti-PD-1	October 2019	Oral FMT capsules + anti-PD-1	Microbiota composition, CD8 TCR diversity, NK cell changes (98)
NCT04924374	Advanced lung cancer on immunotherapy	June 2021	FMT from healthy donors/long-term survivors	Safety and efficacy (ORR, CRR) linked to microbiome modulation (99)
NCT05032014	Liver cancer on anti-PD-1	September 2021	Probiotic *Lactobacillus rhamnosus* (M9)	Microbiota modulation to enhance ICI response (99)
NCT05094167	NSCLC on PD−1+platinum	October 2021	Probiotic cocktail (V9)	Gut microbiota and ICI efficacy/toxicity (100)
NCT04909034	NSCLC on pembrolizumab	May 2021	Fermented soybean extract (MicrSoy−20)	Microbiome-targeted nutraceutical adjunct to PD−1 therapy (101)
NCT04552418	Solid tumors on dual ICIs	September 2020	Resistant starch (prebiotic)	Modulating microbiota to reduce ICI-induced colitis and improve outcomes (101)
NCT03817125	Melanoma on PD−1 therapy	January 2019	Oral microbiome product SER−401 vs placebo	Defined consortia to augment anti-PD−1 antibody efficacy (102)
NCT05462496	Pancreatic cancer	July 2022	Gut microbiome modulation in combination with oncologic therapy	Changes in tumor immune activation and microbiota (79)

*EU FP7-funded; no ClinicalTrials.gov registration.

This table presents selected examples spanning direct antibody approaches (IgY, monoclonal antibodies), microbiome modulation via fecal microbiota transplantation (FMT), and defined bacterial consortia in the context of immune checkpoint inhibitor (ICI) therapy and infectious diseases. The selection is illustrative rather than exhaustive, emphasizing diversity of intervention modalities and disease contexts. FMT, fecal microbiota transplantation; ICI, immune checkpoint inhibitor; IgY, immunoglobulin Y (avian-derived antibodies); mAb, monoclonal antibody; RCT, randomized controlled trial.

### 4.1 *Clostridioides difficile* infection and recurrence

Recurrent *C. difficile* infection is a prototypic condition where repeated eradication attempts with antibiotics are counterproductive: each course further depletes commensals, weakens colonization resistance, and predisposes to relapse (1–3, 26). Bezlotoxumab, a human mAb targeting toxin B, exemplifies an explicitly functional silencing strategy. By neutralizing toxins without targeting vegetative bacteria, it reduces mucosal damage, inflammation, and recurrence risk when given alongside standard-of-care antibiotics. In the MODIFY I (NCT01241552, registered November 2010) and MODIFY II (NCT01513239, registered June 2012) trials (n = 2,655), bezlotoxumab reduced *C. difficile* recurrence by 38% relative to placebo (10.0% vs. 26.6% in the combined modified intent-to-treat population), with a favorable safety profile (71). Microbiome analyses indicate that bezlotoxumab does not substantially worsen antibiotic-induced community disruption and may facilitate more stable recovery by preventing repeated inflammatory insults (58, 71).

From the eradication vs. coexistence perspective, *C. difficile* recurrence prevention illustrates that it can be enough to disarm toxin-mediated pathology while allowing microbial communities, including residual *C. difficile*, to be re-integrated into a more stable ecosystem. Eradication-focused alternatives, such as prolonged or pulsed vancomycin regimens, may reduce short-term burden but at the cost of further dysbiosis and resistance (1–3). Antibody-mediated functional silencing offers a path to break the recurrence cycle without escalating collateral damage.

### 4.2 Acute enteric infections

In acute bacterial gastroenteritis, conventional management relies on supportive care and, in selected cases, antibiotics aimed at eradicating the pathogen, an approach that risks exacerbating dysbiosis and prolonging pathogen shedding (1–3). Antibody-based interventions offer an alternative by reducing disease severity without sterilizing the gut.

IgY preparations directed against ETEC, *Salmonella*, and *Vibrio cholerae* have shown efficacy in animal models and small-scale human trials, predominantly open-label or controlled feeding studies with limited sample sizes, reducing diarrheal severity, stool frequency, and pathogen load when administered prophylactically or therapeutically (41, 47, 64, 72). Large, randomized, placebo-controlled trials with microbiome-resolved endpoints have not yet been conducted for any oral IgY indication.

In high-risk scenarios, severe cholera or systemic invasion, eradication may remain the primary goal, and antibodies serve as adjuncts to antibiotics. Toxin-neutralizing antibodies against cholera toxin or Shiga toxin limit fluid loss and systemic complications while antibiotics reduce viable counts (63, 65, 77). Here, functional silencing reduces acute morbidity, whereas eradication addresses transmission and carriage. This combination highlights how coexistence-based control and clearance can be layered depending on clinical priorities.

### 4.3 Helicobacter pylori

*H. pylori* colonization is traditionally treated with combination antibiotic regimens aiming for eradication, but resistance and microbiome disruption are increasing concerns (1–3). IgY antibodies against *H. pylori* adhesins and urease have demonstrated reduced colonization, gastric inflammation, and urease activity in animal models and small, mostly uncontrolled clinical studies (41, 46–48, 68, 69). A randomized trial evaluating oral IgY-enriched egg yolk against *H. pylori* (NCT06973889, registered January 14, 2025) has now been completed, but RCT data have not yet been published.

Given the strong association between persistent colonization and gastric malignancy, current guidelines still favor eradication. Adjunctive IgY could reduce antibiotic burden, resistance selection, and relapse without replacing clearance (1, 2, 41). This context underscores that functional silencing may be most useful here as a bridge or adjunct rather than a standalone alternative.

### 4.4 Aquaculture and animal health

Outside human therapeutics, IgY-based functional silencing has been widely deployed as a non-antibiotic strategy in animal health and aquaculture, providing a natural experiment in long-term coexistence-based control. Oral IgY targeting enteric pathogens in poultry, swine, and fish reduces disease burden, improves growth performance, and lowers reliance on in-feed antibiotics, primarily through adhesion blockade, agglutination, and toxin neutralization (41, 46–48, 62–64, 72, 78, 79). Microbiome analyses, though typically confined to culture-based or low-resolution 16S surveys rather than shotgun metagenomics, generally show reduced pathogen abundance with preservation of overall diversity and beneficial taxa (41, 46–48, 62).

These applications demonstrate that large-scale, long-term deployment of antibody-based functional silencing can be feasible and ecologically compatible in complex microbial ecosystems. While extrapolation to human disease must be cautious, animal and aquaculture data support the notion that targeting virulence and colonization functions can control disease and reduce antibiotic use without precipitating catastrophic community collapse.

### 4.5 Emerging horizons

The principles of antibody-based functional silencing may extend beyond enteric infections. Microbiome composition modulates responses to immune checkpoint inhibitors (80, 81), and dysbiosis characterizes metabolic syndrome, IBD, and autoimmune conditions (5, 24, 25). However, no antibody-based interventions, whether mAbs, IgY, or engineered IgA, have been tested in any of these indications, either preclinically or clinically. The conceptual appeal is clear, but feasibility remains entirely hypothetical, and significant challenges, including identifying actionable microbial targets and demonstrating durable benefit in complex, multi-pathobiont conditions, must be addressed before clinical translation can be considered. These applications represent future research horizons rather than near-term therapeutic strategies.

Antibody-based functional silencing can also be positioned alongside other precision microbiome-engineering technologies. Bacteriophage-based approaches share the central logic of selectively targeting pathogenic bacteria while preserving overall community structure, and phage-microbiota dynamics are increasingly recognized as important modulators of gut health and disease (82). Phages and antibodies offer complementary precision: phages can self-amplify and reduce pathogen biomass, whereas antibodies disarm virulence functions without necessarily reducing viable counts. Both face analogous evolutionary challenges, phage resistance and antibody escape, reinforcing the rationale for combination and multi-target strategies. Viewing these modalities together situates antibody-mediated silencing within the broader movement toward next-generation, precision microbiome engineering.

More broadly, the principle that modifying microbiome composition or function can influence host physiology now extends well beyond infectious disease. Fecal microbiota transplantation is being explored in neurodegenerative and other gut-brain-axis disorders, reflecting growing recognition that microbiome-directed interventions can affect systemic and extraintestinal processes through neuroimmune and metabolic signaling (83). Emerging evidence on redox signaling and neuroimmune communication provides additional mechanistic context for how microbiome-directed therapies may exert systemic effects. While antibody-based functional silencing and FMT differ mechanistically, both illustrate the therapeutic potential of targeted microbiome modulation, and the functional silencing paradigm may ultimately find applications in these broader contexts.

## 5 Benefits, limitations, and evolutionary risks

### 5.1 Functional silencing versus eradication

The central question for gut-directed anti-infective therapy is not simply whether antibodies “work,” but how their mode of action compares with classical eradication in terms of ecological cost and clinical durability. Through the mechanisms detailed in Section 3, antibodies attenuate virulence and epithelial access while often allowing organisms to persist at lower, clinically tolerable loads (37, 40, 45, 58, 72). In preclinical and early clinical studies, pathogen-specific IgY or mAbs have reduced diarrheal severity, toxin-mediated damage, and pathogen shedding, yet exerted relatively modest effects on overall alpha-diversity and SCFA-producing commensals compared with antibiotics (Ramirez et al., 2020; Diraviyam et al., 2014), consistent with the microbiome-sparing profile demonstrated for bezlotoxumab (Section 4.1).

Antibody-based strategies do not necessarily replace eradication; they offer a way to decouple control of pathogenic functions from wholesale depletion of microbial biomass, enabling tailored combinations in which the choice between coexistence and clearance is driven by host status, ecological context, and therapeutic goals.

### 5.2 Precision and its limits

Antibody-based functional silencing is inherently antigen-specific. mAbs can discriminate between strains and even subclonal variants, while polyclonal IgY preparations target multiple virulence factors on a defined pathogen without recognizing closely related commensals (3, 40–42, 58, 63, 64). This contrasts sharply with antibiotics, dietary interventions, or FMT, which act at the level of metabolic pathways or entire communities (1–3, 26–28).

However, many dysbiosis-associated conditions involve diffuse community-level alterations rather than a single dominant culprit (5, 24, 25). In such settings, pathogen-specific antibodies may be insufficient unless combined with interventions that address the broader ecological context, diet, prebiotics, or defined microbial consortia. Functional redundancy within microbial communities means that silencing one virulence factor or taxon may be compensated by another unless antigen selection and cocktail design explicitly anticipate this.

Antibody-based approaches are therefore most powerful when key pathogenic circuits can be localized to specific antigens or taxa, while community-level editing remains the domain of FMT, consortia, and environmental modulation. An integrated strategy can use antibodies to “pin down” critical pathobiont functions within a broader ecological intervention.

### 5.3 Evolutionary dynamics, resistance, and the “time bomb” question

Eradication and functional silencing impose fundamentally different evolutionary pressures. Antibiotics exert strong lethal selection, creating a high gradient for resistance mutations and horizontal gene transfer; once resistance arises, it often disseminates across species and contexts (1–3). Antibodies impose a more nuanced, non-lethal selection pressure. By blocking adhesion, toxins, or motility, they reduce the fitness of virulence-expressing variants without necessarily threatening organism survival (4, 35, 37, 40, 45, 52). This may slow the emergence of classical resistance, particularly when polyclonal IgY or multi-epitope mAb cocktails are used, because escape would require simultaneous mutations at multiple binding sites (41, 45, 46, 63, 64, 72, 73). However, functional silencing is not evolution-proof.

Bacteria can evade antibody recognition through well-characterized mechanisms. In uropathogenic *E. coli*, the *FimH* adhesin dynamically shifts between high- and low-affinity conformations; antibodies specific to one conformational state can be shed when the protein transitions to the other, a form of non-mutational immune evasion (84). Phase-variable expression of fimbriae and flagella, whereby entire surface structures are stochastically switched on or off, is widespread among Enterobacteriaceae and could enable pathobionts to transiently escape adhesin- or flagellin-targeted antibodies (85). *H. pylori* provides a direct example: its outer membrane proteins undergo extensive recombination-driven antigenic variation, enabling escape from antibody recognition over chronic infection (86). These mechanisms suggest that single-antigen strategies may face rapid evasion, reinforcing the rationale for multi-epitope cocktails.

The theoretical framework of “imperfect immunity” predicts that non-sterilizing interventions, those that reduce disease without eliminating the pathogen, can diminish selection against virulent variants (87). Under this model, experimentally validated for Marek’s disease virus in poultry (88), interventions that suppress virulence without killing the organism may allow more virulent strains to circulate, provided they retain a transmission advantage. Whether this applies to gut pathobionts under antibody-mediated silencing is untested, but it identifies a concrete risk: chronic functional silencing could select for pathobionts with enhanced intrinsic virulence or alternative pathogenic mechanisms not covered by the antibody cocktail.

The central concern, the “time bomb” hypothesis, is whether incremental evolutionary changes during prolonged coexistence can restore virulence, converting a disarmed pathobiont back into a threat. The concern is grounded in precedent: *Streptococcus pneumoniae* populations underwent serotype replacement following polysaccharide conjugate vaccination, with non-vaccine serotypes expanding to fill the ecological space (89). While serotype replacement reflects antigenic escape from vaccine-induced immunity rather than antibody-mediated silencing per se, the underlying principle, that selective pressure on one set of determinants can drive compensatory diversification in others, is directly applicable. Longitudinal microbiome-resolved trials and experimental evolution studies in gnotobiotic or defined community models will be essential to determine when functional silencing yields durable control and when it simply delays evolutionary rebound.

These evolutionary risks are not merely cautionary; each suggests a concrete, translatable mitigation strategy. First, because single-antigen targeting is vulnerable to conformational switching, phase variation, and antigenic drift, antibody products should be designed as multi-epitope cocktails directed simultaneously at structurally independent and functionally essential targets (e.g., combining anti-adhesin, anti-toxin, and anti-flagellar specificities), so that escape would require improbable simultaneous changes at several conserved sites (84–86). Polyclonal IgY is intrinsically advantageous in this respect, as it recognizes multiple epitopes per antigen. Second, targeting conserved, fitness-critical structures, toxin active sites, conserved adhesin pockets, or essential enzymes such as urease, raises the fitness cost of escape and narrows the mutational space available to the pathobiont (61, 67). Third, the risk of compensatory virulence predicted by the imperfect-immunity framework can be monitored directly: longitudinal surveillance of pathobiont virulence-gene expression, toxin titers, and antigen sequence (for emergence of escape variants), combined with microbiome-resolved readouts of diversity and colonization resistance, would provide an early-warning “coexistence signature” (Section 6.3). A rise in virulence-gene expression or the appearance of antigen-loss variants would signal incipient escape and trigger a switch to eradication (87, 88). In this way, the evolutionary boundary central to our framework becomes operationally testable: multi-epitope design and conserved-target selection reduce the probability of escape, while biomarker surveillance detects it early enough to intervene. Embedding these measures into trial design (Section 6.2) would allow the safety of coexistence to be defined empirically rather than assumed.

### 5.4 Delivery, economics, and equity

mAbs are expensive to develop and manufacture, and systemic administration requires hospital infrastructure (40, 43, 60). While their precision may justify costs in severe infections, deploying mAbs broadly for chronic microbiome modulation may be economically challenging.

Oral delivery mitigates many of these concerns. IgY can be produced at scale from egg yolks at low cost, without terminal animal sacrifice, and exhibits favorable gastrointestinal stability (41, 46–48, 62). IgY does not engage mammalian Fc receptors or complement, reducing inflammatory complications and facilitating chronic use (41, 48). Local delivery reduces systemic exposure and simplifies regulatory pathways, potentially enabling food-grade formulations (41, 47, 62).

Compared with live biotherapeutics, antibody preparations are chemically defined, simplifying quality control and safety oversight. Achieving equitable access will depend on scalable production platforms, stability-optimized formulations, and cost-sharing mechanisms.

These advantages must be weighed against several limitations specific to IgY. Although orally administered IgY is largely confined to the gut lumen and is poorly immunogenic by the enteral route, repeated or high-dose administration carries a theoretical risk of sensitization, and parenteral exposure or compromised barrier integrity could elicit anti-IgY responses or hypersensitivity in susceptible individuals; egg-allergic patients represent an obvious contraindication (41, 46). As a polyclonal product derived from immunized hens, IgY is also subject to batch-to-batch variability in titer, affinity, and epitope coverage, which complicates standardization and regulatory characterization relative to monoclonal antibodies (47, 48). Manufacturing considerations, including flock health and biosecurity, consistency of immunization protocols, downstream purification, cold-chain requirements for some formulations, and the need for robust potency assays, must be addressed for clinical-grade production. Affinity maturation is limited compared with hyperimmune mammalian sera, and gastric acid or proteolytic degradation, though mitigated by IgY’s intrinsic stability, may still necessitate enteric formulation or co-administration with acid suppression. Acknowledging these constraints is essential for a realistic appraisal of IgY’s translational potential.

These considerations carry particular weight for low- and middle-income countries (LMICs), where enteric infections impose their greatest burden and where antimicrobial resistance is accelerating (90). The attributes of IgY align well with LMIC implementation needs: production from immunized hens requires relatively modest infrastructure, costs are low compared with monoclonal antibodies, and oral or food-grade formulations avoid the need for cold-chain-dependent parenteral delivery and trained personnel (41, 47). IgY could in principle, be produced regionally, reducing dependence on imported biologics, and could be deployed prophylactically in high-risk settings or therapeutically to reduce diarrheal severity and antibiotic use. Realizing this potential will require locally validated production standards, demonstration of cost-effectiveness against existing oral rehydration and antibiotic regimens, and integration into existing public health frameworks. Positioned this way, antibody-based functional silencing is not only a precision strategy for well-resourced settings but a potentially scalable, affordable tool for global enteric disease control.

## 6 Clinical prospects and future directions

### 6.1 Clinical positioning: when to eradicate, when to silence

Eradication remains non-negotiable in certain contexts: patients with profound immunosuppression (neutropenia, hematologic malignancy, post-transplant states), disrupted barriers (severe mucositis, extensive inflammatory ulceration), or ongoing systemic infection face high translocation risk, and rapid bactericidal therapy is justified despite ecological cost (5, 23, 91). During outbreaks of highly transmissible enteric pathogens where public health priorities demand reduction of shedding, eradication may be the primary goal. Even in these settings, antibody-based strategies can contribute as adjuncts, neutralizing toxins while antibiotics clear organisms, or providing prophylactic protection during temporary immunosuppression. The adjunctive use of bezlotoxumab (Section 4.1) already validates this model.

Coexistence-based control is more naturally suited to conditions where disease is driven by discrete virulence factors rather than organism presence, where preserving colonization resistance is critical, and where repeated antibiotic courses cause escalating harm. Recurrent *C. difficile* infection, chronic *H. pylori* management alongside triple therapy, and prophylaxis in agricultural settings all fit this profile.

**Figure 2:**
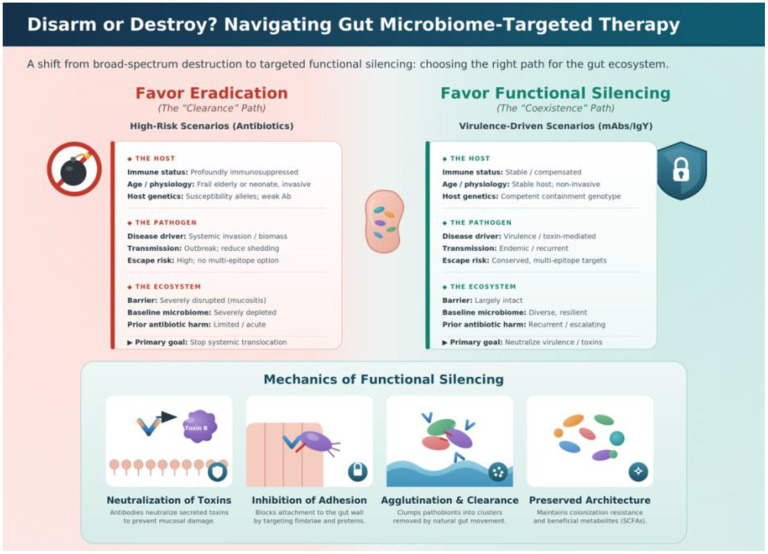
A decision framework for choosing between eradication and functional silencing in gut microbiome-targeted therapy. The diagram organizes the clinical decision along a continuum spanning two therapeutic poles: eradication (left, clearing the organism through bactericidal intent) and functional silencing (right, disarming virulence while preserving the community). Eight determinants are grouped into three domains: the host (immune status and barrier integrity, age and physiology, host genetics), the pathogen (disease driver, transmission and public-health priority, escape potential), and the ecosystem (baseline microbiome diversity and resilience, cumulative antibiotic harm). For each determinant, conditions favoring eradication are shown on the left and those favoring functional silencing on the right. Factors are weighed jointly rather than applied as strict thresholds; any single high-risk host or pathogen factor (for example, profound immunosuppression) can mandate eradication regardless of other inputs. Most patients fall between the poles, where a combination or sequential strategy, layering functional silencing onto reduced-intensity clearance under biomarker-guided surveillance, is most appropriate. A severely depleted community may require ecological restoration (diet, prebiotics, or defined consortia) alongside antibody-mediated silencing. mAb, monoclonal antibody; IgY, immunoglobulin Y.

Beyond immune status and barrier integrity, several patient-level factors are likely to influence suitability for a coexistence-based approach. Age is a key consideration: neonates and infants possess an immature, low-diversity microbiome and developing mucosal immunity, while elderly patients exhibit reduced microbial diversity, immunosenescence, and higher rates of comorbidity; both extremes may shift the risk-benefit balance toward eradication in invasive disease, yet both also suffer disproportionately from antibiotic-induced dysbiosis and may benefit most from microbiome-sparing strategies in non-invasive contexts (1–3). Host genetic variability, particularly polymorphisms in innate immune sensing (e.g., *NOD2*, TLR pathways) and secretor status (*FUT2*), shapes both baseline microbiome composition and the mucosal antibody response, and may predict who can durably contain a disarmed pathobiont (92). Finally, baseline microbiome composition itself, diversity, the integrity of colonization resistance, and the relative abundance of the target pathobiont are likely to be decisive determinants: a resilient, diverse community is better positioned to keep a functionally silenced pathobiont in check, whereas a severely depleted community may require ecological restoration alongside antibody-mediated silencing (4, 5). Stratifying patients by these factors will be essential to define in whom coexistence is both safe and sufficient (**Figure 2**).

### 6.2 Designing trials that test the paradigm

Relatively few studies have explicitly compared eradication-focused versus functional silencing regimens. Future clinical trials should test this contrast directly through head-to-head or add-on designs in indications such as recurrent *C. difficile* or specific enteric infections. One approach: (A) standard-of-care antibiotics alone; (B) reduced-intensity antibiotics plus toxin- or adhesion-targeting antibodies; (C) antibody-based functional silencing with minimal or no antibiotics in carefully selected, low-risk patients. Primary endpoints would include recurrence and serious adverse events, while key secondary endpoints would incorporate microbiome metrics (alpha- and beta-diversity, SCFA levels, pathobiont relative abundance), colonization resistance assays, and resistance emergence. Trial stratification by host factors (immune status, barrier integrity) and microbiome baseline state (enterotype, diversity, pathobiont burden) will be needed to understand in whom coexistence is safe and sufficient. Longitudinal follow-up should monitor evolutionary responses, antigen loss variants or compensatory virulence, that might erode the benefits of functional silencing.

### 6.3 Biomarkers for safe coexistence

Implementing a coexistence strategy demands reliable biomarkers to distinguish stable functional silencing from smoldering risk. Candidates include microbiome composition and function metrics (restoration of SCFA-producing taxa, absence of persistent Proteobacteria blooms, recovery of gene richness), host response markers (normalization of fecal calprotectin, reduced epithelial damage markers), and pathobiont activity readouts (toxin levels, virulence gene expression, metabolite profiles indicating whether pathogenic circuits remain silenced despite ongoing colonization). Combining these with clinical outcomes could define “coexistence signatures”, patterns in which pathobionts are present but their pathogenic functions remain durably suppressed. To date, no validated thresholds have been established in the context of deliberate pathobiont coexistence, and their development should be a priority for early-phase clinical studies.

### 6.4 Integration and regulatory considerations

Antibody-based functional silencing will rarely stand alone. It is more likely to be integrated into multi-modal regimens including diet, prebiotics, probiotics, FMT, or defined consortia. Regulatory frameworks will need to accommodate combination products. Comparative health-economic analyses will be necessary to determine where high-cost mAbs, lower-cost IgY, or engineered IgA are most appropriate, particularly relative to FMT and small-molecule drugs.

## 7 Conclusion

The evidence reviewed here challenges the reflexive equation of therapeutic success with pathogen eradication. In the gut, where most clinically relevant “pathogens” are context-dependent pathobionts embedded within a complex ecosystem, mucosal antibody biology reveals an alternative: functional silencing, selectively attenuating virulence and barrier disruption while preserving community architecture and colonization resistance. Monoclonal antibodies, IgY, and engineered IgA extend this evolved logic to therapeutic contexts, with clinical validation in *C. difficile* recurrence prevention and preclinical promise across enteric infections.

As this review has argued, the choice between the two paradigms is not absolute but contextual: the same pathobiont may warrant eradication in one patient and disarming in another, depending on immune competence, mucosal barrier status, and whether pathology stems from the organism’s mere presence or from specific virulence functions. Defining the boundary between these indications, and determining when coexistence remains safe versus when it becomes an evolutionary “time bomb”, constitutes the field’s central unresolved challenge.

Meeting this challenge will require three advances: (i) clinical trials that directly compare eradication-focused and coexistence-oriented regimens with microbiome structure, function, and resistance evolution as prespecified endpoints; (ii) composite “coexistence signatures”, integrating pathobiont activity readouts, diversity metrics, and host inflammatory markers, that distinguish durable silencing from smoldering risk; and (iii) scalable, affordable platforms for oral antibody delivery, particularly IgY and engineered IgA, that can be integrated into multi-modal microbiome therapeutics. If these conditions are met, disarming and coexisting with pathobionts may become an accepted, evidence-based complement to eradication in gut-targeted therapy.
